# Supplementary material for: Diversity and evolution of computationally predicted T cell epitopes against human respiratory syncytial virus
Source: PLoS Comput Biol. 2023 Jan 10;19(1):e1010360. doi: 10.1371/journal.pcbi.1010360 (PMC9870173; doi:10.1371/journal.pcbi.1010360)
Supplement: S1 Text — Table A: Number of computationally predicted conserved RSV T cell epitopes and experimentally identified RSV T cell epitopes. Table B: Conservation of experimentally validated conserved MHC class I epitopes peptides in RSV major surface proteins in subsampled dataset. Table C: Conservation of experimentally validated conserved MHC class II epitopes peptides in RSV major surface proteins in subsampled dataset. (DOCX) [file pcbi.1010360.s001.docx]

**Supplementary Materials**

# Diversity and evolution of computationally predicted T cell epitopes against Human Respiratory Syncytial virus

Jiani Chen ^[1][2][3][4]^, Swan Tan ^[1][3][4][5],^ Vasanthi Avadhanula ^[6]^, Leonard Moise ^[3][7]^, Pedro A Piedra ^[6]^, Anne S De Groot ^[3][7]^, Justin Bahl ^[1][2][3][4][5][8] *^

* Corresponding author email: [justin.bahl@uga.edu](mailto:justin.bahl@uga.edu)

**This file includes:**

Table A to Table C

**Other Supplementary Materials for this manuscript include the following:**

Supplementary Figure 1 to Supplementary Figure 8

Supplementary Data File 1

**Table A: Number of computationally predicted conserved RSV T cell epitopes and experimentally identified RSV T cell epitopes.**

| **MHC allele** | **RSV**  **protein** | **No. of computationally predicted conserved epitopes^a^** | **No. of experimentally identified epitopes^b^** | **Computationally predicted conserved epitopes with experimental validation** | **Experimentally identified epitopes with computational identification** |
| --- | --- | --- | --- | --- | --- |
| class I | F | 77 | 20 | 12 | 19 |
|  | G | 44 | 4 | 3 | 4 |
| class II | F | 31 | 46 | 18 | 30 |
|  | G | 7 | 4 | 2 | 3 |

1. The conserved epitopes are identified with at least 60% presence across all RSV-A or RSV-B sequences that are publicly available.
2. The number of experimentally identified epitopes include RSV peptides that are positive in MHC class I/ class II ligand assays from the IEDB database after removing duplicates.

**Table B: Conservation of experimentally validated conserved MHC class I epitopes peptides in RSV major surface proteins in subsampled dataset ^a^**

| **Subgroup** | **Protein** | **Epitope address** | **Epitope sequence ^b^** | **Binding HLAs ^c^** | **Conservation ^d^** | Conservation in subsampled dataset ^e^ | **Number of human matches ^f^** | **Epitope id in IEDB** |
| --- | --- | --- | --- | --- | --- | --- | --- | --- |
| RSV-A & RSV-B | F | 45-53 | **LSALRTGWY** | A0101 | 99.55%(A) & 74.24%(B) | 99.42%(A)& 64.61%(B) | 1 | 158982 |
|  |  | 140-148 | **FLLGVGSAI** | A0201 | 99.59%(A) & 97.98%(B) | 99.62%(A)& 99.05%(B) | 0 | 156869 |
|  |  | 250-258 | **YMLTNSELL** | A0201, A2402 | 99.59%(A) & 99.33%(B) | 99.43%(A)& 99.52%(B) | 0 | 156979 |
|  |  | 272-280 | **KLMSSNVQI** | A0201 | 66.64%(A) & 96.08%(B) | 51.43%(A)& 96.91%(B) | 3 | 156902 |
|  |  | 273-281 | **LMSSNVQIV** | A0201 | 66.56%(A) & 96.08%(B) | 51.43%(A)& 96.91%(B) | 1 | 156915 |
|  |  | 449-457 | **TVSVGNTLY** | A0101 | 99.75%(A) & 99.33%(B) | 99.43%(A)& 99.29%(B) | 0 | 97017 |
| RSV-A | F | 10-18 | AITTILAAV | A0201 | 84.69% | 75.89% | 3 | 156844 |
|  |  | 111-119 | LPRFMNYTL | B0702 | 91.18% | 91.40% | 0 | 158975 |
|  |  | 170-178 | ALLSTNKAV | A0201 | 99.67% | 65.56% | 2 | 156847 |
|  |  | 383-391 | NIDIFNPKY | A0101 | 95.86% | 94.84% | 0 | 159045 |
|  | G | 25-33 | FISSCLYKL | A0201 | 99.26% | 98.60% | 0 | 158759 |
|  |  | 61-69 | FIASANHKV | A0201 | 82.08% | 78.20% | 0 | 158751 |
| RSV-B | F | 525-533 | IMITAIIIV | A0201 | 89.25% | 83.14% | 0 | 156892 |
|  |  | 540-548 | SLIAIGLLL | A0201 | 97.65% | 97.86% | 5 | 156960 |
|  | G | 25-33 | VISSCLYKL | A0201 | 90.91% | 85.17% | 0 | 158759 |
|  |  | 61-69 | FIISANHKV | A0201 | 99.02% | 98.43% | 0 | 158751 |

1. This table contains putative MHC class I epitopes that have already been experimentally validated in publications. Only putative Class I epitopes that have positive results in MHC Class I Ligand Assays with the same computationally predicted binding HLAs are listed in the table.
2. Epitope sequences that are conserved in both RSV-A and RSV-B are in bold.
3. HLAs that have the top 1% binder scores in EpiMatrix for epitope sequence.
4. The conservation is evaluated by the presence of epitope peptides across all RSV-A or RSV-B sequences that are publicly available (only epitope sequences with at least 60% conservation are shown in the table).
5. The conservation in the subsampled dataset is evaluated by the presence of epitope peptides across subsampled RSV-A or RSV-B sequences by isolation years and isolating WHO regions.
6. Count of human peptides found in the search database. JanusMatrix was used to search human epitopes that are predicted to bind to the same allele as the RSV epitope and share TCR-facing contacts with the RSV epitope.

**Table C: Conservation of experimentally validated conserved MHC class II epitopes peptides in RSV major surface proteins in subsampled dataset ^a^**

| **Subtype** | **Protein** | **Epitope address** | **Epitope sequence ^b^** | **Conservation ^c^** | |  | | Conservation in subsampled dataset ^d^ | |  | | **Number of human matche ^e^** | **Epitope id in IEDB** |
| --- | --- | --- | --- | --- | --- | --- | --- | --- | --- | --- | --- | --- | --- |
| RSV-A | F | 29 - 44 | TEEF**YQSTCSAVS**KGY | 98.53% |  | | 99.73% | |  | | 3 | | 956680 |
|  |  | 50 - 70 | TGW**YTSVITIELSNIK**ENKCN | 97.75% |  | | 97.90% | |  | | 1 | | 153700 |
|  |  | 167 - 192 | IKSALLSTNKAVVSLSNGVSVLTSKV | 93.14% |  | | 98.28% | |  | | 4 | | 545502 |
|  |  | 218 - 234 | ETVIEFQQKNNRLLEIT | 98.86% |  | | 99.24% | |  | | 3 | | 1087566 |
|  |  | 247 - 268 | VSTYMLTNSELLSLINDMPITN | 98.98% |  | | 99.04% | |  | | 8 | | 99471 |
|  |  | 288 - 310 | IMSIIKEEVLAYVVQLPLYGVID | 98.57% |  | | 98.85% | |  | | 5 | | 99334 |
|  |  | 399 - 418 | KTDVSSSV**ITSLGAIVS**CYG | 99.14% |  | | 99.43% | |  | | 0 | | 545603 |
|  |  | 453 - 470 | GNTLYYVNKQEGKSLYVK | 98.37% |  | | 96.75% | |  | | 1 | | 99691 |
|  |  | 492 - 510 | ISQVNEKI**NQSLAFIR**KSD | 80.32% |  | | 78.78% | |  | | 1 | | 153713 |
|  |  | 543 - 560 | AVG**LLLYCKARSTPV**TLS | 79.26% |  | | 77.82% | |  | | 6 | | 153641 |
|  | G | 19 - 43 | TLNHLLFISSCLYKLNLKSIAQITL | 93.13% |  | | 92.40% | |  | | 8 | | 1087567 |
| RSV-B | F | 29 - 44 | TEE**FYQSTCSAVS**RGY | 99.78% |  | | 99.73% | |  | | 3 | | 956680 |
|  |  | 50 - 70 | TGW**YTSVITIELSNIK**ETKCN | 93.95% |  | | 96.78% | |  | | 1 | | 153700 |
|  |  | 192 - 218 | VLDLKNYINNQLLPIVNQQSCRISNIE | 83.43% |  | | 85.25% | |  | | 4 | | 153636 |
|  |  | 247 - 268 | LSTYMLTNSELLSLINDMPITN | 98.54% |  | | 99.46% | |  | | 8 | | 99471 |
|  |  | 399 - 418 | KTDISSSV**ITSLGAIVS**CYG | 98.88% |  | | 99.73% | |  | | 0 | | 545603 |
|  |  | 453 - 470 | GNTLYYVNKLEGKNLYVK | 98.77% |  | | 98.93% | |  | | 0 | | 99691 |
|  |  | 492 - 510 | ISQVNEKI**NQSLAFIR**RSD | 97.42% |  | | 78.78% | |  | | 1 | | 153713 |
|  |  | 543 - 560 | AIGL**LLYCKAKNTPV**TLS | 94.96% |  | | 95.17% | |  | | 4 | | 153641 |
|  | G | 51 - 74 | STSLIIAAIIFIISANHKVTLTTV | 94.66% |  | | 92.58% | |  | | 8 | | 158751 |

1. This table contains putative MHC Class II epitopes which share the identical binding groove sequence, which represent the nine-mer frames with the greatest potential to bind Class II HLA (epitope sequences with underlines) of the RSV class II epitopes that have already been experimentally validated in publications. Only the putative Class II epitopes that have positive results in MHC Class II Ligand Assays with the same computationally predicted binding HLAs are shown in the table.
2. Epitope sequences that are in bold indicate sequences are predicted to bind class II HLA and are conserved in both RSV-A and RSV-B.
3. Conservation is evaluated by the presence of epitope peptides across all RSV-A or RSV-B sequences that are publicly available (Only epitope sequences with at least 60% conservation are shown in the table).
4. The conservation in the subsampled dataset is evaluated by the presence of epitope peptides across subsampled RSV-A or RSV-B sequences by isolation years and isolating WHO regions
5. Count of human peptides found in the search database. JanusMatrix was used to search human epitopes that are predicted to bind to the same allele as the RSV epitope and share TCR-facing contacts with the RSV epitope.
