# Supplementary figures and images for: Diversity and evolution of computationally predicted T cell epitopes against human respiratory syncytial virus
Source: PLoS Comput Biol. 2023 Jan 10;19(1):e1010360. doi: 10.1371/journal.pcbi.1010360 (PMC9870173; doi:10.1371/journal.pcbi.1010360)

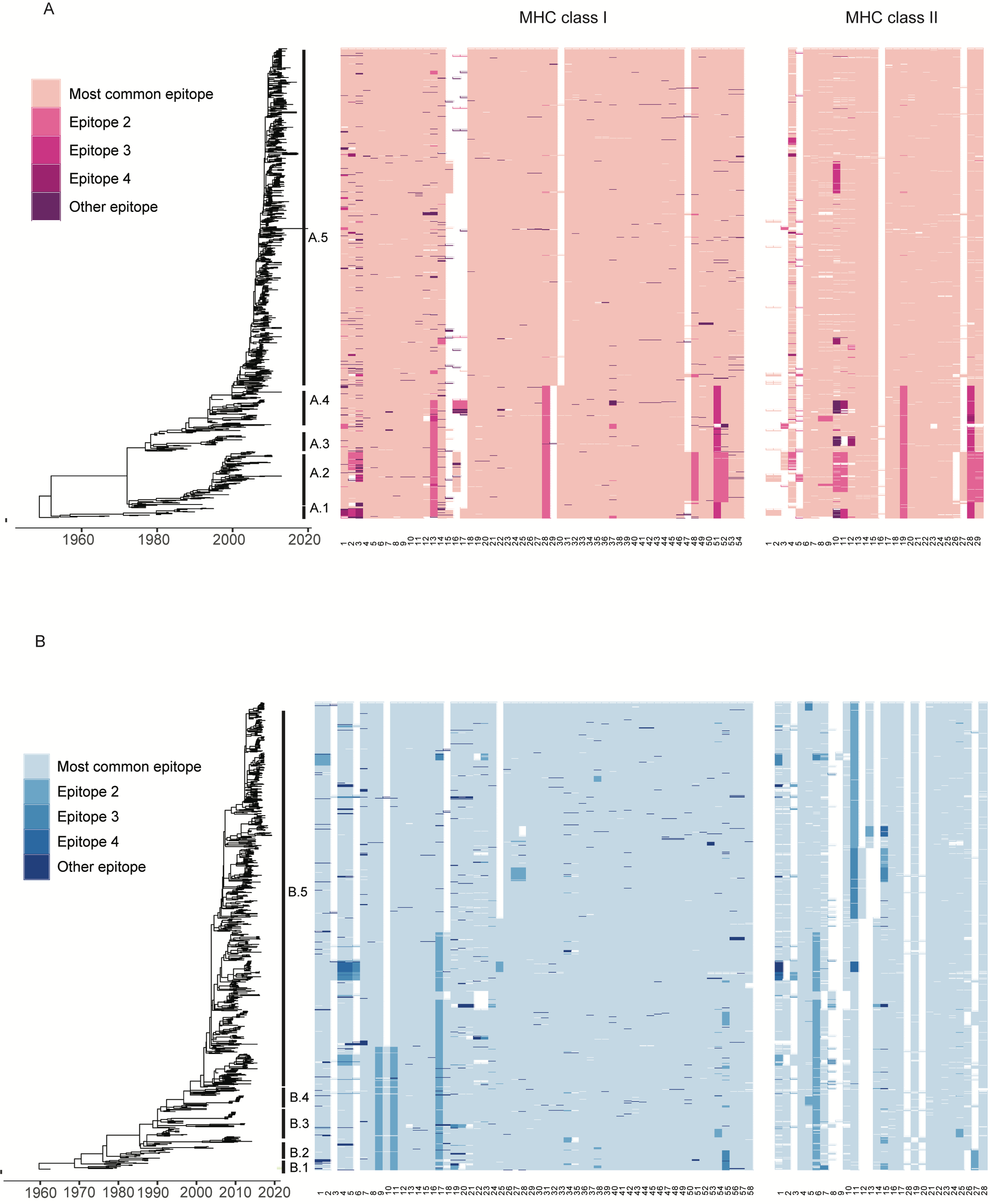

Supplement: S1 Fig — The tree panel on the left is a time-scaled phylogeny build with RSV-A (A) or RSV-B (B) F gene nucleotide sequences using the ML approach. Determined genotypes are labeled on the right with black bars. Each color column on the right side represents the presence of an MHC class I or class II epitope. Only the epitopes that are present in more than 1% of sampled isolates are displayed. The column color indicates different numbers of epitope sequences at the same location. (TIF) [file pcbi.1010360.s003.tif]

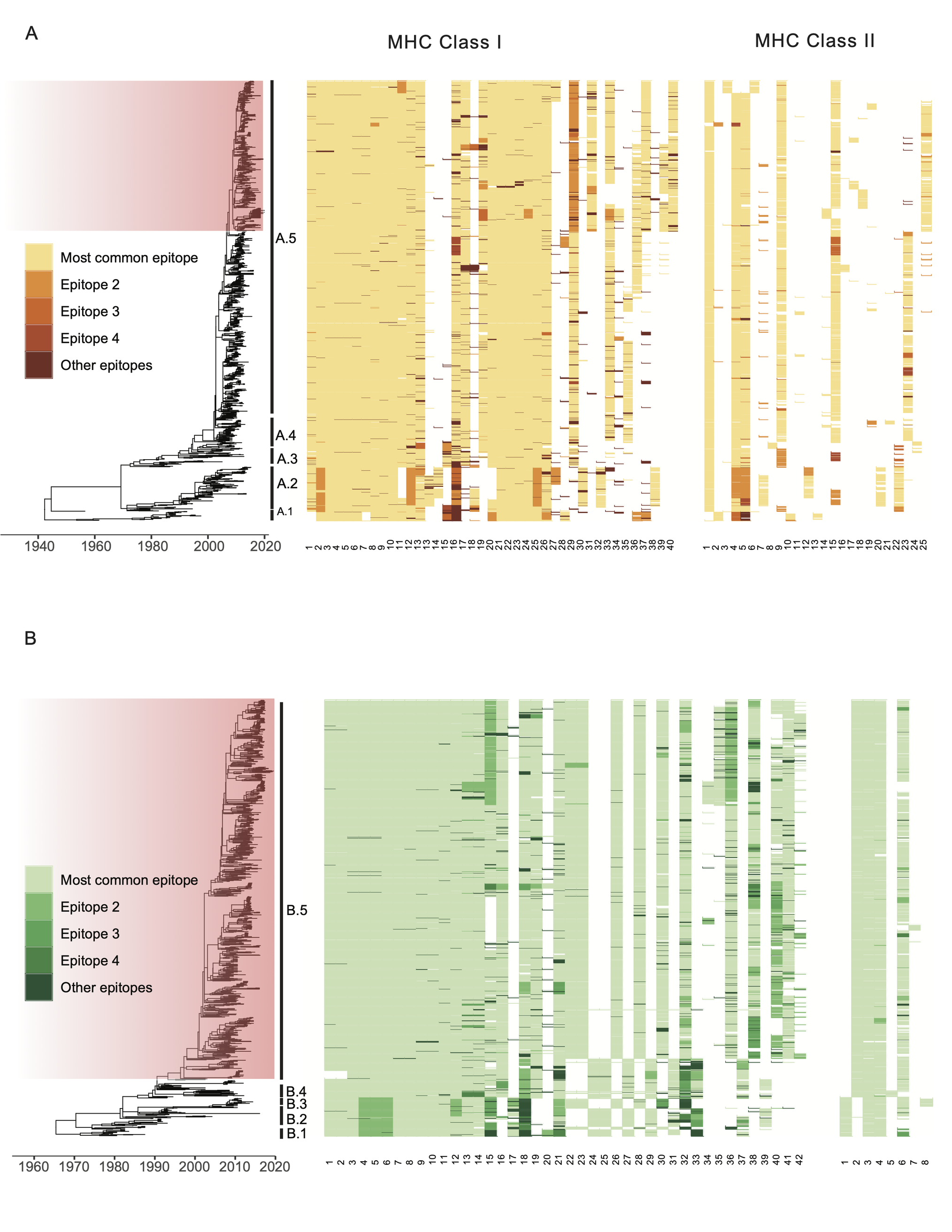

Supplement: S2 Fig — The tree panel on the left is a time-scaled phylogeny build with RSV-A (A) or RSV-B (B) G gene nucleotide sequences using the ML approach. The clades that contain novel 72-nt or 60-nt duplication at the second hypervariable region of G gene were highlighted in red. Determined genotypes are labeled on the right with black bars. Each color column on the right side represents the presence of an MHC class I or class II epitope. Only the epitopes that are present in more than 1% of sampled isolates were displayed. The column color indicates different numbers of epitope sequences at the same location. (TIF) [file pcbi.1010360.s004.tif]

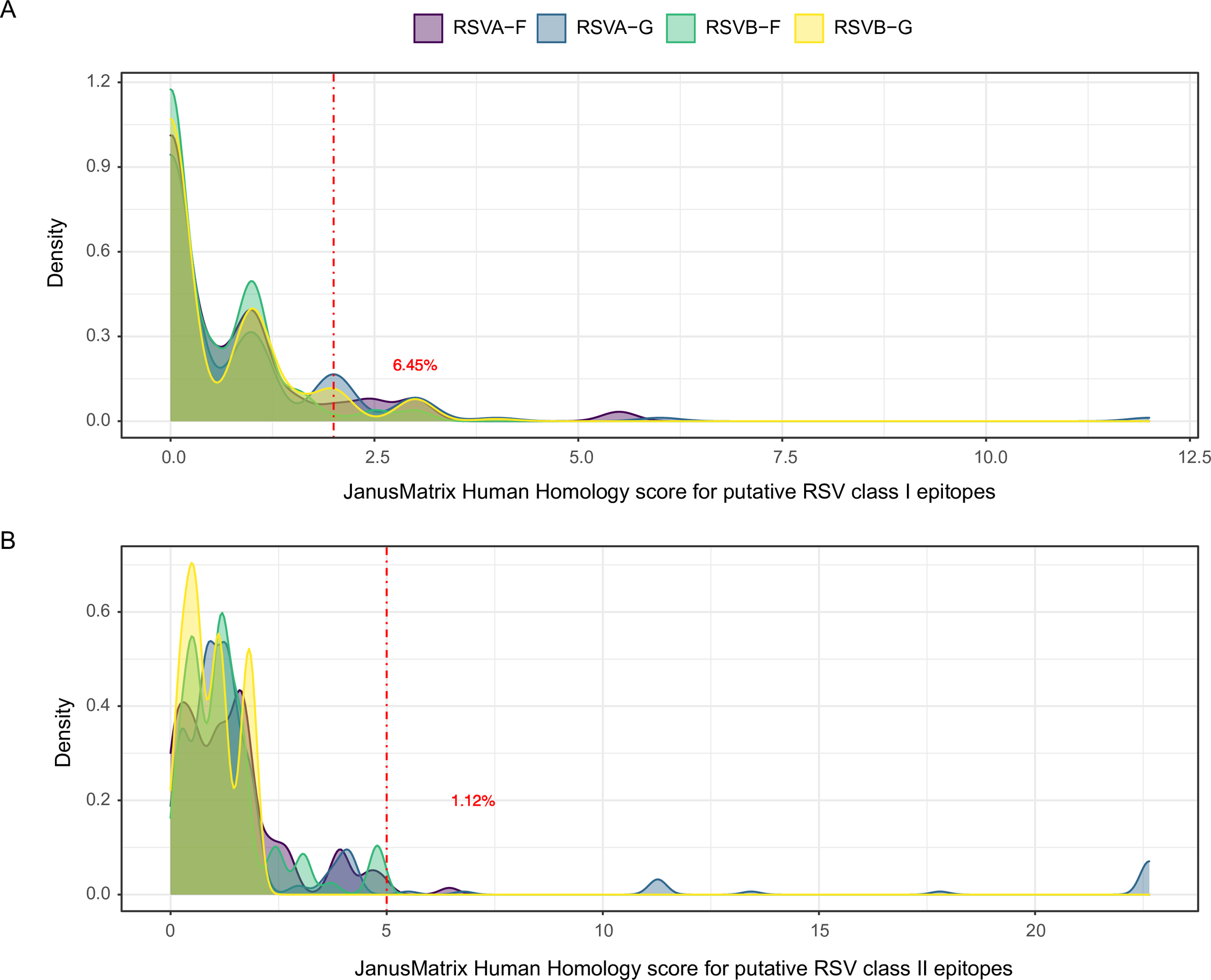

Supplement: S3 Fig — The cross-reactive potential of identified putative T cell epitopes and human host was represented with a JanusMatrix Human Homology score. 6.45% identified putative class I epitopes and 1.12% class II epitopes are cross-conserved on the TCR face with human peptides. (TIF) [file pcbi.1010360.s005.tif]

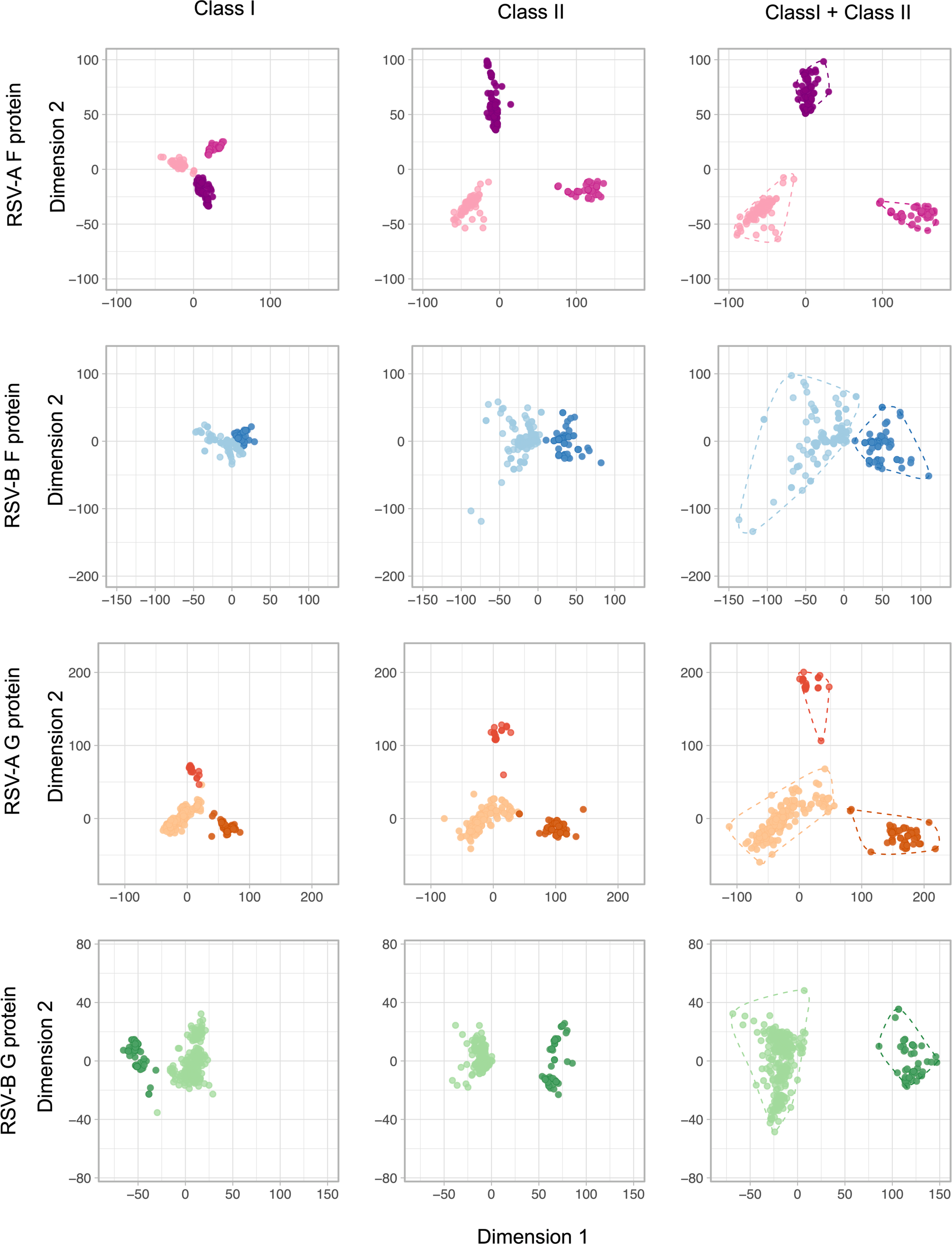

Supplement: S4 Fig — RSV T cell epitope landscapes were built with sequenced-based MHC class I epitope binding prediction (left), MHC class II epitope binding prediction (middle) or combining class I and class II epitope binding prediction (right). Sequences are colored by the epitope cluster determined by epitope landscapes built with combining Class I and Class II epitope prediction (TIF) [file pcbi.1010360.s006.tif]

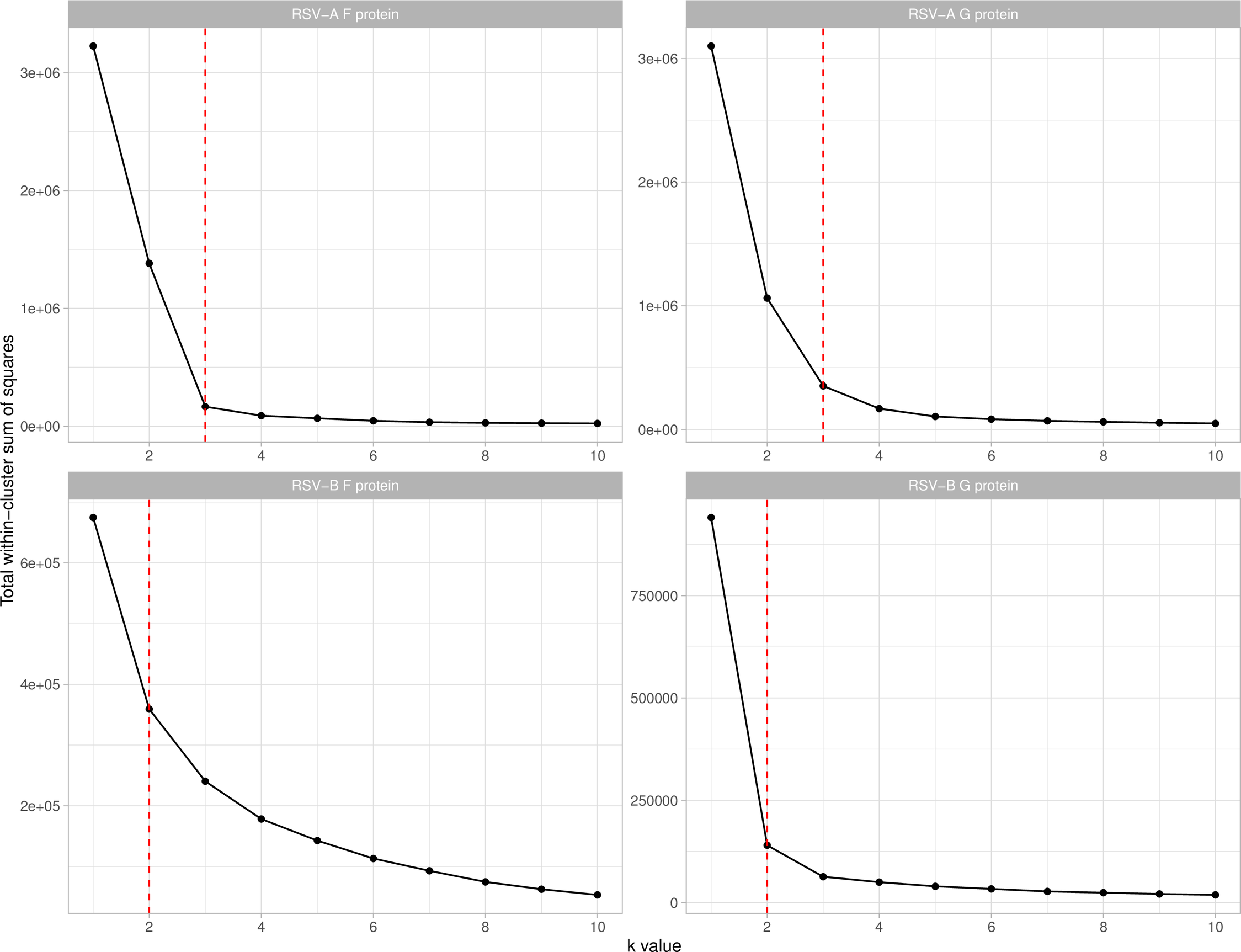

Supplement: S5 Fig — Totals within sum of squares in epitope topographies were calculated after clustering into k (from 1 to 10) groups with k-means. The optimal number of clusters is determined to be 3 in the analysis of RSV-A F and G proteins and is determined to be 2 in the analysis of RSV-B F and G proteins using the Elbow method. (TIF) [file pcbi.1010360.s007.tif]

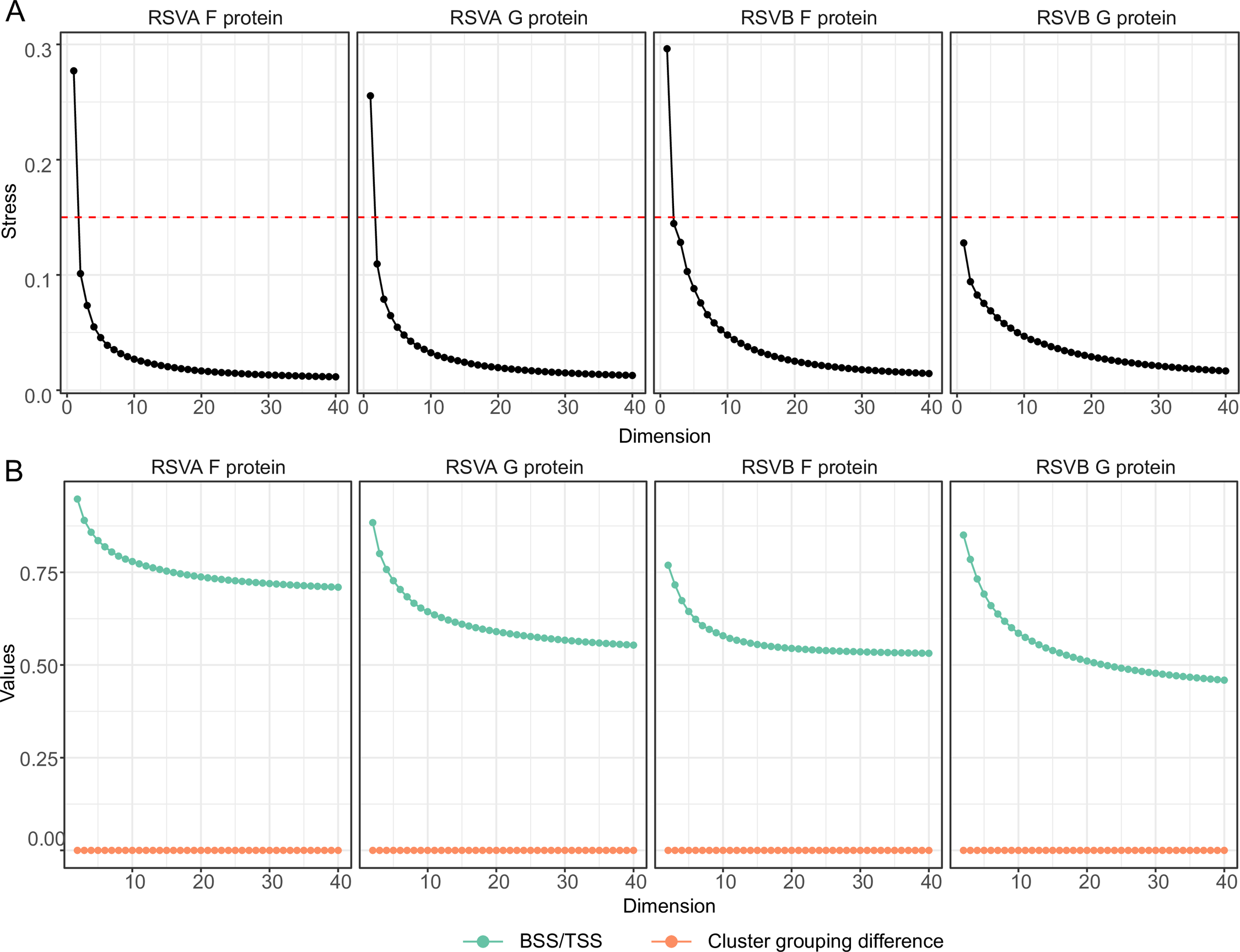

Supplement: S6 Fig — (A) Stress evaluation under the different number of dimensions for RSV distance matrix. Stress less than 0.15 (red dash line) indicates an acceptable precise MDS solution. (B) Performance of k-means clustering under the different number of dimensions, the number of clusters is determined at 2-dimensional space. There is no cluster grouping difference at higher dimensional space (orange). Sum square between clusters /sum square of total differences (BSS/TSS) measures indicates the total variance in the data is explained well under higher dimensional space (green). (TIF) [file pcbi.1010360.s008.tif]

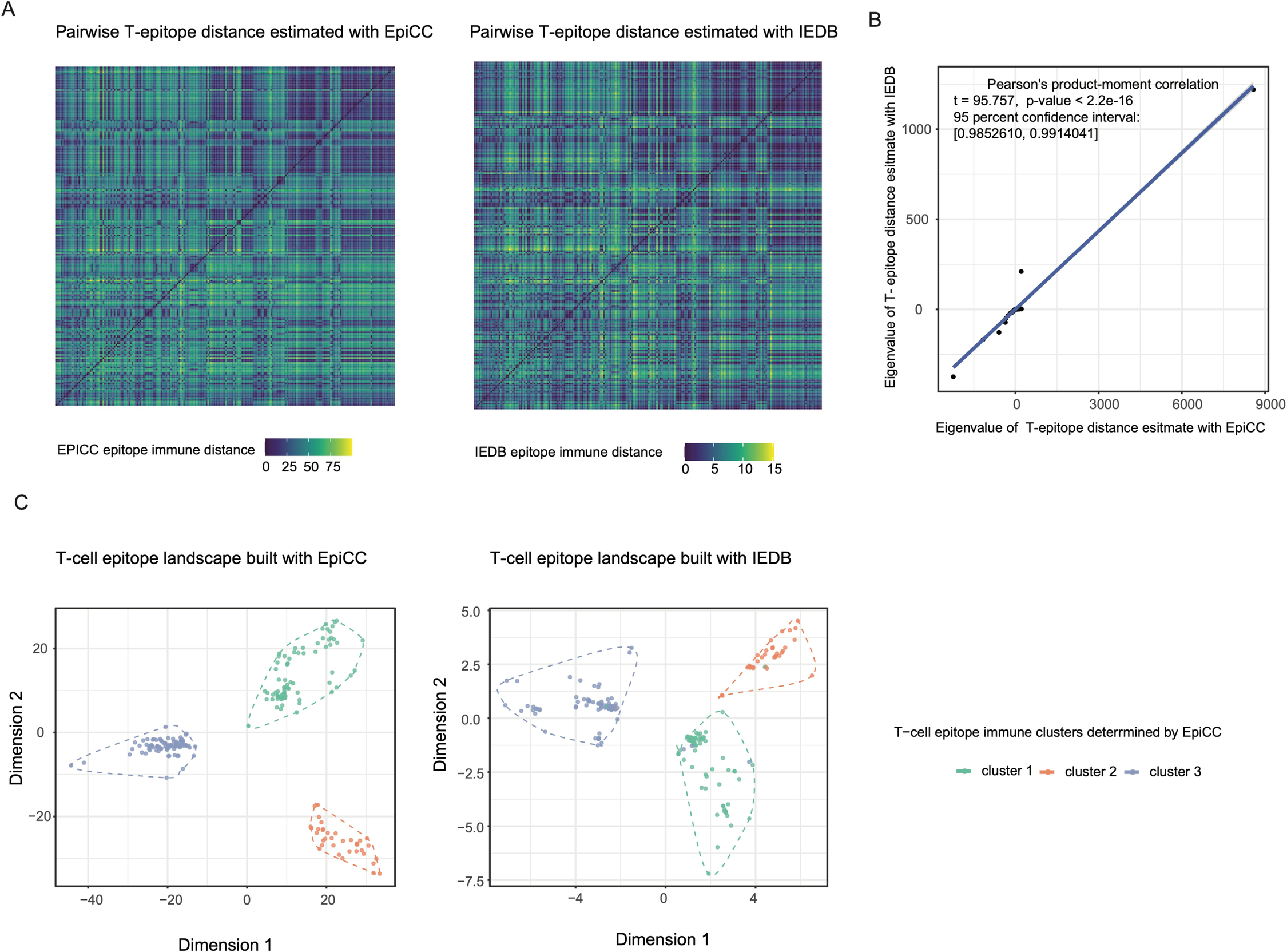

Supplement: S7 Fig — Validation is performed with MHC class I epitope binding prediction of RSV-A F protein. (A) Heatmaps for pairwise MHC class I epitope distance estimated in iVAX toolkits or calculated with custom python scripts using MHC class I molecule binding prediction that is implemented in IEDB. (B) Eigenvalues for each sequence are calculated from pairwise distance matrices using “RSpectra” package in R. The Pearson correlation test significantly supports a non-zero correlation between T cell epitope distance estimated with EpiCC and T cell epitope distance estimated with IEDB. (C) T cell epitope topographies are built with pairwise epitope distances estimated from EpiCC or IEDB. Both methods resulted in a similar cluster pattern for the CD8 T cell epitope profile of RSV-A F protein. (TIF) [file pcbi.1010360.s009.tif]

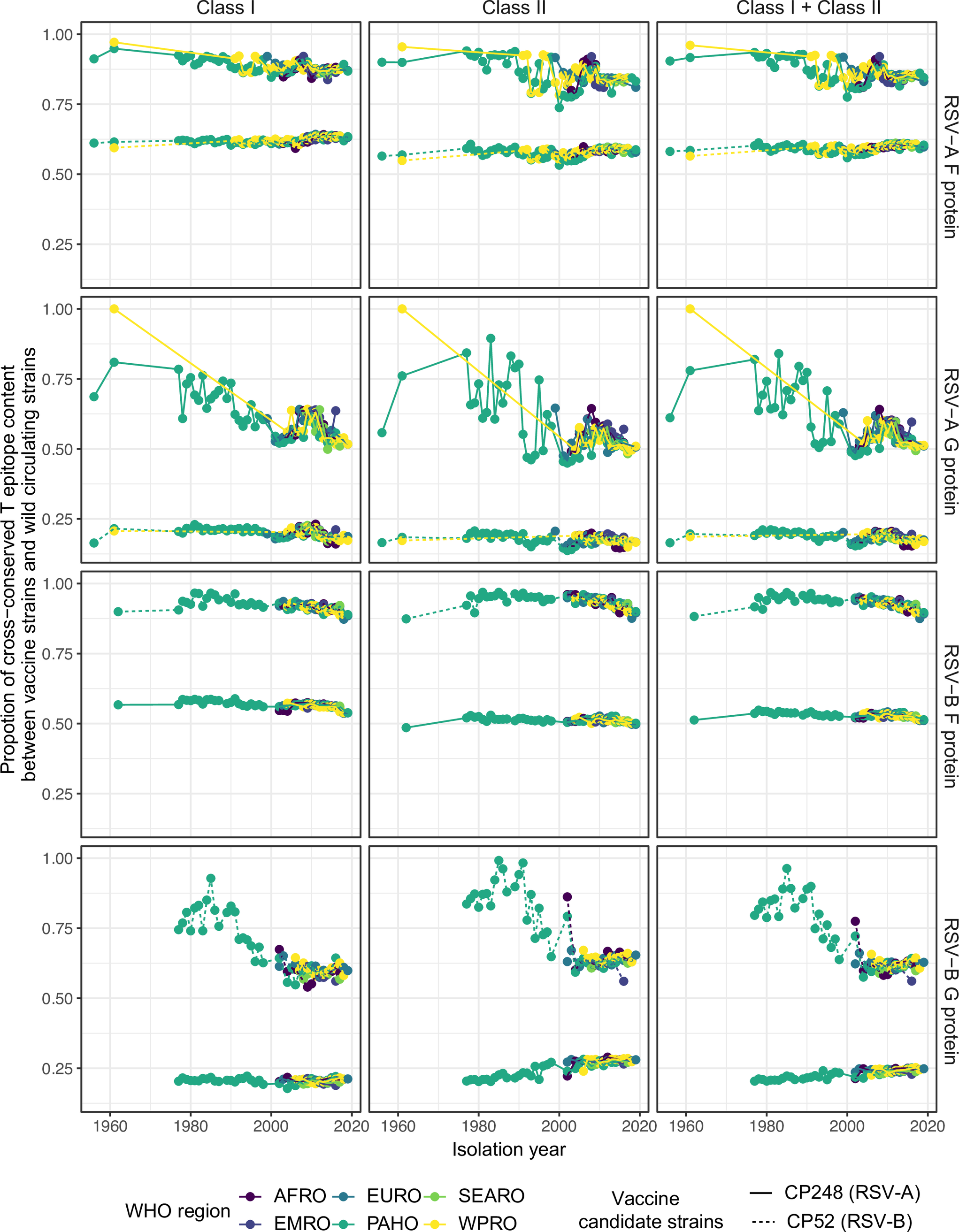

Supplement: S8 Fig — RSV-A and RSV-B major surface protein sequences were grouped by isolation year and 6 isolated WHO regions, African Region (AFRO), Region of the Americas (PAHO), South-East Asia Region (SEARO), European Region (EURO), Eastern Mediterranean Region (EMRO) and Western Pacific Region (WPRO). The proportion of cross-conserved T cell epitope content between vaccine strains (CP248 or CP52) and wild circulating strains in different isolation years and different WHO regions were represented. (TIF) [file pcbi.1010360.s010.tif]
