# Supplementary material for: Diversity and evolution of computationally predicted T cell epitopes against human respiratory syncytial virus
Source: PLoS Comput Biol. 2023 Jan 10;19(1):e1010360. doi: 10.1371/journal.pcbi.1010360 (PMC9870173; doi:10.1371/journal.pcbi.1010360)
Supplement: Attachment — Submitted filename: Chen[CompBio]_RTR_1116.pdf [file pcbi.1010360.s011.pdf]

Friday, Nov 18, 2022

Dr. Feilim Mac Gabhann and Dr. Jason Papin,
Editors-in-Chief,
PLOS Computational Biology,

Thank you for your invitation to re-submit our manuscript. We would also like to thank our reviewers for taking the time to thoroughly review the manuscript and make constructive suggestions.

In response to comments, we modified the methods section adding more detail and addressing concerns. First, we clarified the definition of cross-conserved T cell epitopes and added more detail to the section on how we compute T cell epitope distance between RSV strains. Second, we clarified that the T cell epitope landscapes are constructed based on peptide-MHC allele prediction and therefore are not sensitive to the T cell epitope thresholds we chose. We also add a new method section to provide more details on JanusMatrix algorithm used in human homology analyses. To respond to the reviewers' concerns on data and code sharing, we provide alternative scripts that could reproduce the T cell epitope landscape from raw RSV sequence data via GitHub. We also expand our introduction to make a clearer and stronger argument why studying RSV T cell epitope evolution is important.

We believe that our manuscript is much improved by responding the reviewer inputs. We look forward to the outcome of your assessment. Please find the point-by-point responses to all comments below.

Yours Sincerely,
Justin Bahl PhD, Jiani Chen

We would like to thank the reviewers for their thoughtful comments. We have responded to all concerns and believe our manuscript has been improved.

Response to reviewer 1

The paper by Chen et al presents an analysis of T cell epitope evolution in RSV. Though an interesting study, the results remain much more descriptive than conclusive. Moreover, a number of issues are not clear and therefore it is rather difficult to re-produce the results presented in this paper.

1. The paper makes use of a non-publicly available tool
Response: Thank you for noting this. (1) EpiVax tools were used to perform some of the T cell epitope predictions in this manuscript, which enables the processing of a large number of protein sequences. The tools are available for academic collaborations. For example Giorgetti et al, Clin. Immunol 2021. [1], Tan et al, Vaccines, 2021 [2]. (2) We reproduced our results with publicly available T cell epitope prediction tools (NetMHCpan), and those results are available in the original main text in lines 270-274: *"We used the NetMHCpan method* [3] *to predict T cell epitopes and perform the same landscape reconstruction using MHC class I binding predictions for RSV-A F protein. Our analysis showed a consistent clustered pattern of RSV T epitope profile on the landscape regardless of T cell epitope prediction method (Supplementary Figure 7)."* **The scripts are available on GitHub, as we mentioned in the method section lines 527-531. "***We apply the Equations (1) to re-calculate T cell epitope immune distance with customized Python scripts (available at https://github.com/JianiC/RSV_Epitope/tree/master/NetMHCpan_reproduce) using MHC binding prediction results that are generated from publicly available T cell epitope prediction tool, netMHCpan EL 4.1 methods in the Immune Epitope Database (IEDB)."*
(3) The key difference between EpiVax Tools and netMHCpan is that the EpiVax tool allowed us to process and analyze a larger dataset in order to translate our results to population and ecological infectious disease dynamic scales.

2. Are only the epitopes presented in Table I and II are used to make MDS maps? How many epitopes were predicted by the method but not (yet) validated experimentally.
Response: Thank you for your question. The answer to your first question is No, we built MDS maps with predicted peptide binding affinity of HLA class I and class II supertype alleles across RSV strains. We did not need to identify the specific epitopes at this stage. The epitopes in Table I and II were identified in this study and their binding was confirmed in previous publications, which supports the accuracy of the T cell epitope prediction. In addition to these epitopes, we also identify sets of putative epitopes (Supplementary Figure 1 and Supplementary Figure 2) with similar high MHC binding potential. Experimental characterization of the remaining identified peptides was beyond the scope of our study.
We have edited the main text in lines 234-236 as follows for clarification: *"We performed a pairwise comparison of T cell epitope content between RSV strains using in silico predicted peptide-HLA allele binding affinity. The pairwise T cell epitope distances were calculated using the algorithm reported in this study (Equation 1)."*

To answer your second question, we have listed the number of computationally predicted conserved T epitopes and experimentally identified T epitopes in Supplementary Table 1.

**MHC allele**	**RSV protein**	**No. of computationally predicted conserved epitopes**	**Experimentally identified epitopes**	**Computationally predicted conserved epitopes with experimental validation**	**Experimentally identified epitopes with computational identification**
class I	F	77	20	12	19
	G	44	4	3	4
class II	F	31	46	18	30
	G	7	4	2	3

3. Vaccine section: what is the definition of being conserved? If an epitope still binds to its MHC, is it then a conserved epitope? T cell binding can still be totally lost.

Response: Thank you for the question. We defined conserved T cell epitope in the Methods section in the main text (lines 502 - 510):

"The Epitope Content Comparison (EpiCC) algorithm, which is implemented in iVAX was used to compare T cell epitope content within each subsampled dataset by evaluating cross-conserved T epitopes (9-mer peptides with identical TCR-facing residues and are predicted to binding to the same MHC allele) content between different virus strains [4]. *We reasoned that epitopes with identical TCR-facing residues (TCRf, position 4, 5, 6, 7, 8 for class I epitopes binding core and 2, 3, 5, 7, 8 for class II epitopes binding core) regardless of differences in their MHC-facing amino acids, which are also predicted to bind to the same MHC allele, are more likely to induce cross-reactive memory T cells (These epitopes are called cross-conserved T cell epitopes)."*

We further clarify the definition of cross-conserved T epitopes in the main text before the method sections (lines 286-289):

"T cell epitopes that are similar between vaccine strains and wild strains (cross-conserved T epitopes, which are defined as epitopes that share identical T cell receptor-facing residues and are restricted by the same alleles [32]) may be responsible for the T cell immune protection of the vaccine"

4. At line 224 it is suggested that visualising T cell epitope evolution via MDS is a new approach. As the authors state it in the discussion, this is not the case.

Response: Thank you for pointing out this ambiguity and we acknowledge the discrepancy. The new application that we refer to is our use of MDS to characterize the T cell immunity profile of globally distributed RSV viruses. We have modified the text as follows (line 232):

"To investigate the evolution of RSV on T cell immunity profiles, we use a multidimensional scaling (MDS) approach to visualize the T cell immunity profile of multiple RSV strains on a landscape."

5. JanusMatrix algorithm is used extensively in the paper, but the reader gets very little information on what this model does.

Response: We have added an explanation in the methods section to describe the JanusMatrix algorithm in lines 462-473:

"Identification of cross-conservation between putative RSV epitopes and human peptides

We also applied analysis of human homology to this study. After identifying putative T cell epitopes sequences in RSV major surface proteins, the JanusMatrix algorithm [5] *was used to assess the potential cross-conservation of T cell epitopes with epitopes restricted by the same HLA alleles in the human proteome (Uniprot-sourced human proteins, UniProt* [6]*). JanusMatrix scans input peptides and takes the 9-mer epitope regions that are identified in EpiMatrix to find the human peptides with a compatible HLA-facing agretope (i.e. the agretopes of both the input peptide and its human counterpart are predicted to bind to the same HLA allele) and the same T cell receptor (TCR) facing epitope to compute as a JanusMatrix Human Homology Score. As defined in retrospective studies, foreign class I epitopes that score greater than 2 and class II epitopes that score greater than 5 may be less immunogenic due to T cell tolerance* [7]*. "*

6. How were the alleles included in this study decided? Why are there only two HLA-B alleles and many more HLA-DRB alleles? Clearly allele choice might have a big impact on the results.

Response: We agree with your point that each individual's HLA type may impact the success of a vaccine and therefore when T cell epitopes are selected for vaccination, they should be representative of a broad selection of HLA. We added the explanation of the allele choice in line 445-447:

"*These are HLA allele supertypes (alleles sharing common binding preferences) that cover the genetic diversity of more than 95% of human populations globally* [8], [9]**"**

From A. Sette and J. Sidney (1999) "We currently estimate that six major supertypes alone account for over 85% of HLA-A genes, and 40% of HLA-B genes. In practical terms, epitopes from just the six most frequent supertypes afford an average population coverage of 99.3% [8]" and from S. Southwood et al (1998): "In conclusion, these data demonstrate that a large set of DR molecules encompassing DRB1*0101, DRB1*0401, DRB5*0101, DRB1*1501, DRB1*0701, DRB1*0901, and DRB1*1302 is characterized by largely overlapping peptide binding repertoires [9]. "

7. The analysis makes use of a lot of thresholds to identify a T cell epitope. How are these thresholds defined? Are the results sensitive to the thresholds.

Response: We used 3 thresholds to identify a potential T cell epitope and have cited the corresponding publications in the main text: (1) HLA binding affinities are expressed as Z scores by the EpiMatrix epitope mapping tool. These scores are computed by (i) computing a binding affinity score based on the known HLA binding motif (9mer peptides) and (ii) normalized to a Z scale by comparing the distribution of binding affinity scores from a random set of peptides with the same length. Previous studies showed that 95% of eluted Class I 9- and 10-mers epitopes had scores above 2.32 [7] **and we, therefore, use this threshold to define Class I epitopes in this study.**

(2) Class II T cell epitopes have been found to cluster [10]**. The ClustiMer tool** [11] **was developed to find Class II T cell epitopes with a cluster score above 10 that are highly likely to be immunogenic in human populations, which was defined retrospectively by evaluating published epitopes and is described in detail in the paper by De Groot et al., 2020** [7]**.**

(3) We use the JanusMatrix tool to explore whether the T cell epitopes identified by EpiMatrix and ClustiMer are potentially cross-conserved with human peptides. Please find details in our response to Reviewer 1 at comments #5. Class I epitopes with Janus score greater than 2 and Class II epitopes with a score greater than 5 are less likely to be immunogenic due to T cell tolerance, which is validated in retrospective and prospective studies [7]**.**

To answer your second question (1) The thresholds described above are used to identify immunogenic epitopes and have been validated in prospective and retrospective studies [7]**. Additionally, we searched the IEDB epitope database to determine if these computationally identified T cell epitopes were related to experimentally validated RSV T cell epitopes and HLA ligands. Since this study was primarily concerned with the evolution of T cell epitope content in RSV over time and was not designed to re-evaluate the thresholds, these previously defined and validated thresholds were accepted as the criteria for epitope selection in our study. (2) MDS map and potential vaccine efficacy evaluation are not sensitive to thresholds. Instead of defining T cell epitopes with a threshold, our equations take predicted MHC-peptide-HLA binding affinity score to evaluate T cell epitopes difference/similarity between RSV strains quantitatively.**

8. Why are the Class I and II epitopes are combined in this analysis?
Response: We combined Class I and Class II epitopes to provide a summary of information for the T cell immunity profile of RSV strains. We also provide MDS maps for class I and Class II epitopes separately in Supplementary Figure 4. Similarly, we assessed vaccine efficacy through Class I and Class II epitopes content separately in Supplementary Figure 7.

9. Both in Table I and II it would be nice to add what kind of experimental validation is found? in vitro binding, elution, T cell response?
Response: Thank you for the suggestions. We have revised the captions of Table 1 and Table 2 accordingly.
The added description of Table 1 in lines 200-202: "*a…. Only putative class I epitopes that have positive results in MHC class I ligand assays with the same predicted binding HLAs are shown in the table.***"**
The original Table 2 captions*:" This table contains putative MHC class II epitopes which share the identical binding groove sequence of the RSV class II epitopes that have already been experimentally validated in publications."*
The revised description of Table 2 in line 215: *"This table contains putative MHC class II epitopes that share the identical binding groove sequence, which represent the nine-mer frames with the greatest potential to bind class II HLA (epitope sequences with underlines), with the RSV class II epitopes that have already been experimentally validated in publications. Only the putative class II epitopes that have positive results in MHC class II ligand assays with the same computational predicted binding HLAs are shown in the table."*

10. Table II: are predicted and validated epitopes are the same or do they only have the same core?

Response: Thank you for your question. Our criteria to include the computationally predict conserved epitopes in Table II is to have the identical sequence with the experimentally validated epitope at the core region (underline regions in Table 2). We have updated the caption of Table 2, please refer to our response to your comments at point 9.

11. Figure 2 caption: indicate what A, B, C and D panels are.
Response: Thank you for your suggestion, we have edited Figure 2 caption in lines 276-284: *"Filled circles indicate RSV F protein isolates or G protein isolates without duplication. Diamonds indicate G protein isolates with gene duplication. (A) Epitope landscapes of RSV major surface proteins are built with MHC class I and class II epitope content comparison across different strains. T cell immunity clusters are determined with k-means method and are used to color the sequenced isolates in the following panels. (B) The corresponding time-scaled phylogenies are reconstructed with the Maximum Likelihood (ML) approach. (C) T cell epitope immune distance and (D) genetic hamming distance from the estimated TMRCA are plotted against the isolated time of each sequence."*

12. Which thresholds are used to make NetMHCpan analysis?
Response: Thank you for your question. We have explained the NetMHCpan analysis in the method section in lines 657 to 661: *"We adapt the Equations (1) to re-calculate T cell epitope immune distance with customized Python scripts (available at https://github.com/JianiC/RSV_Epitope/tree/master/NetMHCpan_reproduce) using MHC binding prediction results that are generated from publicly available T cell epitope prediction tool, netMHCpan EL 4.1 methods in the Immune Epitope Database (IEDB) "* **We did not need to identify the specific epitopes at this stage. Equation (1) takes the predicted peptide-MHC binding affinity of 9-mer peptides from pairs of RSV sequences, therefore, no thresholds are used in our analyses. In the method section in lines 515-523**: *"We use u to represent 9-mer peptides with similar MHCf capable of binding the same MHC alleles but bearing different TCRf (non-cross conserved T cell epitopes) in two wild circulating strains (w_1 and w_2). Because the T cell immune response to virus is directly related to its T cell epitope content, the T cell immune distance (D) between two strains can be represented by the sum of binding probabilities of these unique 9-mer peptides for a set of HLA alleles (Equation 1). $p(u)_a$ is the predicted binding probability of unique 9-mer peptide u against a single class I or class II allele a, that is a member of A, which represents a set of HLA alleles.*

$$D(w_1, w_2) = \sum_{u \in (w1, w2)} \sum_{a \in A} p(u)_a \quad (1)$$

13. The sentence starting on line 78 does not sound correct to me.
Response: We have edited the sentence and added citations in line 78:
The original text:" Due to the recent breakthrough to structural constrain the F protein in the pre-fusion conformation and the development of RSV rodent models, there has been a surge in the number of RSV vaccine candidates undergoing clinical evaluation."

The edited sentence in lines 78 - 80" *Recent developments, including resolution of the F protein* [12] *and development of RSV rodent models* [13] *have contributed to a number of vaccine candidates with novel designs and formulations currently in clinical trials* [14]–[16]*"*

14. Line 232: biding --> binding
Response: Thank you. We have edited accordingly in line 241.

<u>Response to reviewer 2</u>

Chen et al investigate the diversity of RSV from the perspective of T-cell epitopes. These epitopes might be an interesting target for future vaccine development, both because they may provide broad immunity, but also because of the problem of vaccine-induced disease. The authors make use of of-the-shelf bioinformatic tools to find candidate CD4 and CD8 T cell epitopes for common HLA alleles. They filter out epitopes that are similar to human-protein derived peptides and compare the predicted epitopes with experimentally validated ones from IEDB. While I do think the approach can be useful and interesting, I have a couple of concerns that should be addressed. Most importantly, the method section has to be re-written and made much clearer.

Major comments:

1. The introduction was well written and is very informative, except for the last part of the fourth paragraph. I'm not sure what the relevance of cross-conserved epitopes is. I'm not sure what cross-conservation means. The final sentence does not follow naturally from this paragraph. Try making a better argument for why characterizing T-cell epitope profiles is important.
   Response: We appreciate the reviewer's suggestions. We rewrote this paragraph to highlight the importance of T cell epitope profiles of RSV and the cross-conserved section has been removed.
   The original text: "*T cell epitopes are sometimes cross-reactive, which is defined as the recognition of two or more epitope peptide-MHC complexes by the same T cell receptor and these cross-reactive epitopes are predicted to be cross-conserved* [17]. *Prediction of cross-conservation is important for vaccine design because it would be useful to predict protection against a pathogen with different lineages and identify escape variants. Hence, characterizing T cell epitope profiles across different strains can be crucial for RSV vaccine development."*
   New text in lines 107 to 114: *"While highly conserved T cell epitopes in RSV vaccine may not provide complete protection against infection when cross-protective antibody responses are lacking, highly conserved T cell epitopes in the vaccine may still reduce the severity of the illness and limit the spread of the virus. However, amino-acid variation at the T cell epitope level and the potential emergence of novel T cell epitopes of recent RSV circulating strains have been reported* [18]*, and further studies are needed to illustrate the effect of amino acid variations on T cell recognition. Hence, characterizing T cell epitope profiles across different strains is very important to understand RSV evolution and can be important for RSV vaccine development."*

2. Identifying epitopes after a gene duplication event: On page 10 you mention that gene duplication events can "shift" the position of epitopes or create novel epitopes. One could argue that a "shifted" epitope is the same epitope as the original. How is this counted when you compare strains in T-cell antigenic space? Are shifted epitopes identified, or counted as separate? How does this influence the clustering?
Response: Thank you for the questions. As you mentioned, the "shifted" epitope in RSV isolates with G gene duplication has identical amino acids with the epitopes of previous RSV G protein isolates. We explained it in the main text in line 168-170: *"The G gene duplication events in RSV, which are unique gene signatures, can either shift the position of epitopes (locations are different but the amino acids of epitopes are identical to the G protein isolates without duplication) or cause the emergence of novel epitopes."*

These shifted T cell epitopes are not considered as T cell changes in the equation (Equation 1) to compute T cell epitope distance that we introduced in this study. Therefore, these shifted T cell epitopes have no effects on T cell epitope landscapes (MDS). We have described in the main text in lines 243-245 "*We observe that the G gene sequence isolates that contain 72-nt (RSV-A) or 60-nt (RSV-B) duplications clustered together with other sequences instead of forming isolated groups."* **We further added in discussion in lines 348-350 "***We find the RSV strains with G gene duplications can still cluster with previous RSV isolate on the T cell epitope MDS space. Our results highlight the importance of characterizing T cell epitope changes in RSV."*

3. Clustering in low-dimensional space. As MDS is a non-linear dimension reduction method, clustering the data in the low-dimensional "MDS-space" could result in artifacts. For instance, points might cluster together because they are projected together, not because they are actually close in the original "epitope space". This may not be an issue in this case, but I suggest checking this by first clustering the data, and then applying MDS. You might need a clustering method that works directly on the distance matrix (Leiden clustering perhaps?). Alternatively, you could increase the dimension of the MDS space, and use k-means on this higher-dimensional MDS space.
Response: To address the reviewer's concern, we re-evaluated whether applying k-means clustering to classify RSV strains in 2-dimensional space can reflect their T epitope profile in Supplementary Figure 6. (1) We calculate the stress of MDS under different numbers of dimensions (Fig. S6 A). Stress that is less than 0.15 (red dash line) indicates an acceptable precise MDS solution. (2) We also compare the performance of *k-means* clustering with increased dimensions of MDS spaces (Fig.S6 B). Our result indicates increasing the dimensions of MDS-space does not have big effects on *k-means* clustering and data interpretation.
We updated our method section in lines 567-571: "*To evaluate whether applying k-means clustering to classify RSV strains on two-dimensional space can reflect their T epitope profile, we calculated the stress of MDS using the* smacof *package in R* [19]. *We also compare the performance of k-means clustering in different numbers of dimensional spaces and the number of clusters that are determined in 2-dimensional space."*

4. The definition of distance between strains. I don't understand the logic behind equations 1.1 and 1.2. Why is this a good distance measure between strains? I also don't understand the

notation in 1.1. What is p(i)a? Are you multiplying p(i) with a? I thought that a is an HLA allele. You mention that you take z-scores of the probabilities p. At what point does this happen? Can distances be negative? It appears to me that something is wrong here. This has to be explained in a more rigorous manner.
The same holds for equations 2.1 and 2.2. Why switch to a different definition here (why don't you use a single distance measure?). I don't understand what you mean by "T-cell cross-conservation between two epitopes can be represented by a joint probability estimation and therefore …". Why is 2.1 a product, while 1.1 was a sum?
Response: Thank you for your questions. We changed p(i)a to $p(i)_a$ and clarified this in the main text. Yes, a is a single HLA allele, and we intend $p(i)_a$ to represent the binding probability of epitope *i* against an HLA allele *a* that is predicted using a T cell epitope prediction algorithm. We have simplified the equations to measure T cell epitope distance to avoid confusion. The text has been modified as follows:
in lines 515-523: *"We use* u *to represent 9-mer peptides with similar MHCf capable of binding the same MHC alleles but bearing different TCRf (non-cross conserved T cell epitopes) in two wild circulating strains (*w_1 *and* w_2*). Because the T cell immune response to virus is directly related to its T cell epitope content, the T cell immune distance (D) between two strains can be represented by the sum of binding probabilities of these unique 9-mer peptides for a set of HLA alleles (equation 1).* $p(u)_a$ *is the predicted binding probability of unique 9-mer peptide* u *against a single class I or class II allele* a*, that is a member of A, which represents a set of HLA alleles.*

$$D(w_1, w_2) = \sum_{u \in (w1,w2)} \sum_{a \in A} p(u)_a \qquad (1)$$

We use equation (1) to measure T cell epitope differences between two strains, and equations (2.1 and 2.2) to quantify the potential efficacy of vaccine candidate strains, which is related to the probability they induce T cells that can be recalled upon exposure to a wild circulating strain. We modified this section by explaining the use of joint probability to represent the probability for recall of vaccine-induced T cells by a circulating wild strain for two conserved 9-mer peptides:
lines 536-553:"*The capacity for a vaccine to induce a T cell immune response that could be recalled by a wild circulating strain is related to the cross-conservation of the T cell epitopes between the vaccine strain (*v*) and the wild circulating strain (*w*). For each pair of 9-mer peptides* i *(from strain* v*) and* j *(from strain* w*) that are cross-conserved (i.e. bearing identical residues that face the TCR), the probability to recall cross-reactive T cell memory by those two 9-mer peptides via a single HLA allele* a *can be represented by the joint estimation of the binding probability of these two 9-mer peptides (*$p(i)_a * p(j)_a$*)."*

Then we introduced the T cell epitope similarity score calculation using this joint probability estimation:
Therefore, a T cell epitope similarity score (S) between two sequences can be represented by summing the probability to cross-reactive memory T cells by all paired 9-mer peptides that are cross-conserved between vaccine strain (v*) and wild circulating strain (*w*) against a set HLA alleles A (Equation 2.1).*

$$S(v,w) = \sum_{i\in v, j\in w} \sum_{a\in A} (p(i)_a * p(j)_a) \quad (2.1)$$

We further normalized the T cell epitope similarity score between the vaccine strain and wild circulating strain by the maximum T cell epitope similarity score for the vaccine strain in comparison with itself (Equation 2.2):

$$P(v,w) = \frac{\sum_{i\in v, j\in w} \sum_{a\in A} (p(i)_a * p(j)_a)}{\sum_{i\in v, j\in v} \sum_{a\in A} (p(i)_a * p(j)_a)} \quad (2.2)$$

5. Several sets of epitopes are mentioned: predicted epitopes (1%, 5%), then human peptides are filtered out, and then you list a set of experimentally confirmed epitopes. In the end, which set of epitopes is used for the MDS analysis and clustering?
Response: Thank you for your question. The epitopes in Tables I and II were identified in this study and their binding was confirmed in previous publications, which supports the accuracy of the T cell epitope prediction. We built MDS maps with predicted peptide binding affinity of HLA class I and class II supertype alleles across RSV strains and we did not need to identify the specific epitopes at this stage. Reviewer 1 had a similar concern and have more thoroughly responded above. Please refer to our response to Reviewer 1 on comment #2.

Minor comments:

1. In the case of Influenza, some evidence exists that T-cell epitopes are subject to positive selection in humans. For instance Machkovech et al (J. Virol. 2015) compared the substitution rate in epitope sites with that of swine flu, and in Woolthuis et al (ref 32) the number of epitopes appears to decrease with time. It would be interesting to do a similar analysis for RSV. Have you tried this? Perhaps you can at least comment on this.
Response: Thank you for your comments. We suspect RSV may have a different pattern from influenza. Immune selection has been reported as the main driver of influenza evolution and circulation [20]**. In contrast to influenza, local persistence of RSV has been reported and only a few antigenic sites of RSV surface proteins are under selection** [21]**. In our analysis, we observed RSV with similar T cell epitope content can circulate for a long time and different T cell immune clusters can co-circulate. While this is not the focus of our manuscript, we agree it would be interesting to study if RSV T cell epitopes are under positive/negative selection in the future.**

2. You give a list of experimentally verified epitopes. However, are there any epitopes in IEDB that are not present in the predicted dataset? If so, why are they not predicted?
Response: Please refer to our response to Reviewer 1 in comment #2. Most RSV Class I epitopes in IEDB are present in the predicted dataset, while some of them are not conserved among RSV strains. We also find several RSV Class II epitopes in IEDB that are identified as MHC high binder using computational tools, while we do observe some are not present in our predicted datasets (Supplementary Table 1). There are many potential reasons, for example: (1) The MHC restriction of the RSV epitopes in IEDB may not be captured by the MHC supertypes that we used in computational tools. (2) We identify putative class II epitopes based on a predicted high density of MHC class II binding potential, while experimentally characterized RSV T cell epitopes are positive

with single T cell epitope assay. (3) We are lacking detailed information on the experimental protocol of T cell assays used in each study.

3. Line 20: the B-cell response.
Response: Thank you for your suggestion. Both "B-cell" and "B cell" have been used in publications, we keep it consistent throughout the manuscript with "B cell"

4. Line 38: The B-cell epitope
Response: Please refer to our response to your comment # 3.

5. Line 80: add some references? Or the names of some of these studies?
Response: Following your suggestion, we added new references in this sentence. Please find our response to reviewer 1 in comment #13.

6. Line 105-108: I don't understand this sentence. Please re-phrase.
Response: Thank you for raising your concern. The original text:" *Monoclonal antibody tests have demonstrated additional antigenic variability within RSV-A /B antigenic groups and suggest that it may play a role in the ability of RSV to escape immune response and established infections"*
We edited the sentence in line 105: "*Several lines of evidence also suggest antigenic variation may play a role in the ability of RSV to escape immune response and established infections* [22]"

7. Line 112: What does cross-conserved mean?
Response: Thank you for your queries. Following your suggestions in major comment #1, we rewrote this section, and the word "cross-conserved" have been removed. Please refer to our response to Reviewer 2 in comment #1.

8. Line 125: do you mean "Isolation years"?
Response: Thank you for pointing this out. We have corrected it in line 123.

9. Line 133: Dependent on the final order of sections (Intoduction, Results, Discussion, Methods), at this point it is not clear how this score is generated. Perhaps give a one-sentence explanation and mention the +20 significance level.
Response: Thank you for your suggestions, we provide how we calculate protein-level epitope density score and the meaning of +20 in the method section "protein-level immunogenic potential evaluation" of the main text in lines 475-491. Following your suggestion, we add a brief explanation here (in lines 131 to 134):" *The epitope density of RSV surface proteins was evaluated using a normalized epitope density score, which is computed by summing up the predicted peptide-MHC binding score across the protein and normalizing it with the protein length. The score for randomly generated proteins is set to zero and antigens with significant T cell immunogenic potential generally score above +20 on this scale* [7] *(Figure 1A)"*

10. Line 144: You mention that T-cell epitopes and Ab-epitopes overlap, but figure 1B does not show the location of Ab-epitopes. Would it be possible to indicate those in the 3d structures?

Response: Thank you for the suggestions. We have updated Fig. 1B with antigenic sites on the 3D structure of RSV F protein.

11. Fig S1: Is the "dominant" epitope the most common epitope or the epitope with the highest score? Somewhat confusing terminology.
Response: Thank you for raising your confusion. We have modified it to "the most common epitopes" in Fig. S1 and Fig. S2.

12. Line 179: EpiMatrix has not been mentioned before.
Response: Thank you for pointing this out. We mentioned EpiMatrix in the following methods section, and we have edited the sentence accordingly. The original text:" To further determine whether the T cell epitopes defined using EpiMatrix might be immunogenic…"
and we have edited the sentences in line 181:" *To further determine whether the computationally predicted T cell epitopes with high MHC binding potential are immunogenic…."*

13. Line 181: cross-conserved? Do you mean cross-reactive?
Response: Here we perform *in silico* analysis to define putative RSV T cell epitopes that have conserved TCR-facing residues with human peptides (See Tables I and II). We use cross-conserved here because we do not perform T cell assays for these peptides and we also do not include data showing that the T cells of this conservation are reactive. By inference, these peptides may also be 'reactive' but we believe that to call them cross-reactive would be an exaggeration and therefore we prefer the term cross-conserved.

14. Table 1: Conservation is calculated using all available sequences. But did you account for sampling bias for more recent strains? If an epitope is present in a more recent strain, and many similar strains are also isolated, then the conservation is artificially inflated.
Response: We acknowledge the potential sampling bias of the RSV sequences that are currently available, but we assume the RSV genetic surveillance can reflect the real molecular epidemiology of RSV in our analyses. To account for the potential sampling bias for more recent strains, we further evaluate the conservation of the identified T cell epitopes in a subsampled dataset, which is subsampled by isolation year and isolated WHO region (Methods: Subsampling strategy), no big differences were observed. The conservation in the subsampled dataset was included in Supplementary Tables 2 and 3.

15. Line 203: perhaps say "human peptides" as these are (hopefully) not actual epitopes.
Response: Thank you for pointing this out, we changed "human epitopes" to "human peptides" throughout the manuscript.

16. Line 238: Cluster 1 is paraphylectic, which is quite interesting, but might this also depend on the parameters/settings used for clustering. With alternative settings, cluster 1 could perhaps be split into 2 separate clusters that might become monophylectic. Can briefly you comment on this?

Response: Thank you for your question. (1) To confirm the clusters are paraphyletic, we incorporate the isolation date of the sequence to rebuild the phylogenies in Figure 2B, which are rooted by the most recent common ancestors. Cluster 1 is still paraphyletic on this time-scale phylogeny. (2) We comment on this in lines 247-251 *"The F gene cluster 1 is paraphyletic, while clusters 2 and 3 are monophyletic. Cluster 1 is the closest to the ancestral sequence and mapping this group onto the phylogeny shows that this cluster has a basal relationship with clusters 2 and 3 indicating that the phylogenetic divergence occurred prior to epitope drift."*

17. Line 281: Has EpiCC been introduced?
    Response: Thank you for pointing this out. We rewrote this sentence. The original text: *"To quantitatively evaluate whether it might be necessary to include multiple RSV strains to prepare an effective vaccine, two live attenuated RSV strains that are previously considered as vaccine candidates, CP248, a recombinant virus that belongs to subtype A, and CP52, which is a recombinant RSV-B strain, were included in our analysis and compared to wild-type strains using EpiCC."*
    The edited text in main lines 289"*To quantitatively evaluate whether it might be necessary to include multiple RSV strains to prepare an effective vaccine, … were included in our analysis and we evaluated their T cell epitope conservation with different RSV wild circulation strains.***"**

18. Figure 3: This is not a very clear representation of the data. Would a simple bar plot be better?
    Response: Thank you for your suggestion. We provided an alternative visualization of Figure 3 as a line plot in Supplementary Figure 7. The radar plots have the advantages to display the predicted T cell immune protection of vaccine candidates against RSV strains in different categories and we, therefore, chose to keep it in the main text.

19. Line 363: "Possibility" instead of "probability"
    Response: Thank you for your suggestion. We edited accordingly in line 379.

20. Line 371: This paragraph is a bit weak. Can you say what these biases might be? And what are these valuable insights?
    Response: Thank you for your suggestion. The original paragraph:" The lack of experimental data might cause problems in epitope identification. Computational-based T cell epitope landscapes have the potential for bias. But the observed clustered T cell evolutionary pattern in RSV surface proteins provide valuable insights into virus evolution in the aspects of T cell immunity and strain selection for vaccine design.**"**

    We rewrote this paragraph in line 387:" Our current study is limited by lacking experimental validation of T cell epitope prediction. We focus on computationally predicted MHC binding to identify T cell epitopes. Although the strength of MHC binding is the key parameter that determines a peptide's immunogenicity, but not sufficient for a module to be immunogenic [39]. Other aspects associated with pathogen-induced T cell immune response, such as appropriate antigen-processing [40] and T cell receptor recognition [39] are not considered in this study, which might cause bias in the computational-based T cell epitope

landscapes. However, the observed clustered pattern of RSV surface proteins on the T cell epitope landscape in this study reflects the diversity of T cell epitopes within different strains. This finding provides valuable insights into virus evolution in the aspects of T cell immunity and can contribute to the strain selection for vaccine design.*"*

21. Line 423: "Each one overlapping the previous one by one amino acid": surely you mean overlapping by 8 amino acids?
Response: Thank you for raising your concern. Yes, we are talking about scanning each 9mer peptide overlapping by 8 amino acids with a sidling window approach. We have edited the word.
The original text: "*EpiMatrix parsed 9-mer sequence frames (each one overlapping the previous one by one amino acid) from the antigen sequence"*
The edited sentence in line 447: *"EpiMatrix parsed 9-mer sequence frames (each one overlapping the previous one by eight amino acids) from the antigen sequence"*

22. Line 480: What does this mean: "are assumed to be mutually exclusive and uniform"? Please explain this more clearly.
Response: We add an explanation in line 511 "*To simplify the analysis, the binding of 9-mer epitopes within protein sequences are assumed to be mutually exclusive and uniform, which means the T cell immune response of the antigen protein can be represented by summing up all T cell epitopes within the protein sequence."*

23. Line 496: where in the main text are you using this? Also, please explain more clearly what you are doing here (and why)
Response: Thank you for raising your concern. We have explained why we use T cell epitope prediction from netMHCpan EL 4.1 methods to regenerate RSV T cell epitope landscape in the result section in lines269-274: *"There are multiple methods available to predict T cell epitopes* [25]*, which may result in different reconstructed landscapes if there is a systematic bias in the prediction method. We used the NetMHCpan method* [3] *to predict T cell epitopes and perform the same landscape reconstruction using MHC class I binding predictions for RSV-A F protein. Our analysis showed a consistent clustered pattern of RSV T epitope profile on the landscape regardless of T cell epitope prediction method (Supplementary Figure 7)."*

24. Line 521: what was the result of the goodness-of-fit analysis?
Response: Thank you for your question. This was used to justify whether 2-dimensional space can reflect the original T cell epitope distance of RSV virus. We have removed this and rewrote this section. Please refer to our response to Reviewer 2 major comments #3.

25. Line 528: How did you take these insertions into account in the genetic distance?
Response: Thank you for your question. Please find the "calculation of genetic hamming distance" in the methods section in lines 573-579: *"Genetic hamming distance, which is defined as the number of bases by which two nucleotide sequences differ, was calculated by comparing the number of different bases between each sequence in the subsampled datasets."*

The genetic distance calculation can directly reflect the number of nucleotides inserted in gene insertion events.

<u>Response to reviewer 3</u>

The paper is an interesting read and describes an elegant computational analyses that provides novel insights that improve understanding of immunity to RSV. I have some minor questions that may be addressed by the authors to broaden the discussion a bit:

1. The work takes a number of HLA types into account to find relevant epitopes. Considering the diversity of HLA alleles in a population of individuals that will be vaccinated, how would an individual's HLA typing impact the success of a vaccine that has been designed by this computational approach? In other words: does a selected peptide work out well for a large part of the whole population of vaccinees? Or should a wide selection be made from a large pool of different peptides? How do the authors envision such an approach towards vaccine design?
Response: Thank you for your comments. Please refer to our response to Reviewer #1 at point 6. We explain this in the main text in lines 444-446: "*These are HLA allele supertypes (alleles sharing common binding preferences) that cover the genetic diversity of more than 95% of human populations globally* [8], [9]**"**

2. The authors stress the variations among RSV G proteins. However, the RSV G protein contains a central conserved domain that has been shown to provide T-cell immunogenicity. What would such conserved domain mean for predicting T-cell epitopes and immunity to G protein?
Response: Thank you for pointing out this. The conserved domain would be an important component of a multi-epitope vaccine, due to its conservation in multiple strains. In our manuscripts, we also identified highly conserved T cell epitopes of RSV G protein (Table 1 and Table 2). We have further highlighted the importance of conserved T cell epitopes of RSV G protein in discussion in lines 328-331: "*In contrast to the conserved F protein, RSV G protein is reported to be highly variable, we still observed potential conserved T cell epitopes across different strains, which suggests a great interest of G protein conserved domain as potential vaccine target on T cell immune protection"*

3a. Many epitopes are left out of the analysis due to similarity to human sequences, since this would generate tolerance. That is a fair point. However, these epitopes may also actively induce Treg cells that may control excessive T-cell responses causing unwanted inflammation. Can the authors exclude that such 'active control' as means of tolerance may be involved in the overall immune response to RSV? In other words, would neglecting such 'tolerant' epitopes pose a risk for excessive immune responses when regulatory T cells are not allowed to be activated sufficiently?
Response: Thank you for asking this interesting question. The exclusion of human-like epitopes is meant to eliminate T cell epitopes that are recognized by circulating natural regulatory T cells (these nTregs presumably are trained on epitopes that are already existed in the human proteome). We agree the immune response to RSV also likely includes induced tolerance by Tregs to RSV epitopes, which would not be eliminated by this step. In natural infection, the effect of these epitopes may be to reduce inflammation. Since T

effector (T helper and CTL) epitopes are the focus of vaccine development, we did not consider these epitopes for our analysis. We do believe that the role of these epitopes during infection should be experimentally validated. However, that is currently beyond the scope of this study.

3b. Were epitopes in tables 1 and 2 indeed validated to be tolerated experimentally, where indicated? And was immunogenicity of these sequences checked and never observed?
Response: Thank you for your comments. Table 1 and Table 2 contain published epitopes that were experimentally validated in MHC binding assay, the corresponding IEDB accession numbers are provided. Please find our response to Reviewer 1 in comment #9. However, the tolerogenic of these sequences were not determined to our knowledge. In our study, we provide our human homology JanusMatrix results in the "Number of human matches" columns of Table 1 and Table 2 using where a higher score suggests that T cells recognizing that epitope may exhibit a bias toward immune tolerance.

4. In figure 1B it might be helpful the reader to point antigenic sites ø and II in the 3D-structures of pre-F and post-F.
Response: Thank you for the suggestions, we have updated Fig. 1B with antigenic sites on the 3D structure of RSV F protein.

5. Figure 2A describes 'Tenaus toxin'. Is this Tetanus toxin?
Response: Thank you for pointing this out. We have corrected the typo in Fig. 1A.

Have the authors made all data and (if applicable) computational code underlying the findings in their manuscript fully available?
The PLOS Data policy requires authors to make all data and code underlying the findings described in their manuscript fully available without restriction, with rare exception (please refer to the Data Availability Statement in the manuscript PDF file). The data and code should be provided as part of the manuscript or its supporting information, or deposited to a public repository. For example, in addition to summary statistics, the data points behind means, medians and variance measures should be available. If there are restrictions on publicly sharing data or code —e.g. participant privacy or use of data from a third party—those must be specified.
Reviewer #1: **No:** The authors are using a non-publicly available tool and their analysis is hard to reproduce because not all details are given. There is some code available in github but I wander if one can use it without having the predictions.
Response: Thank you for raising this concern. We provide all of the setting we used with the T cell epitope prediction toolkit iVAX in the sections. We also provide the codes that we used to perform T epitope prediction with publicly available T cell epitope prediction tools "NetMHCpan" via GitHub (https://github.com/JianiC/RSV_Epitope/tree/master/NetMHCpan_reproduce). The RSV T cell epitope landscape in this manuscript can be reproduced start from RSV sequences without having T cell epitope prediction.

Reviewer #2: **No:** The authors use publicly available data, but did not share any of their scripts.

Response: We have deposited the scripts via GitHub. Please find it in our acknowledgements. All scripts used in this manuscript can be accessed through [https://github.com/JianiC/RSV_Epitope].

Reviewer #3: Yes

Reference:

[1] S. I. Giorgetti *et al.*, "Development of highly stable and de-immunized versions of recombinant alpha interferon: Promising candidates for the treatment of chronic and emerging viral diseases.," *Clin. Immunol.*, vol. 233, p. 108888, Dec. 2021, doi: 10.1016/j.clim.2021.108888.

[2] S. Tan *et al.*, "Quantifying the Persistence of Vaccine-Related T Cell Epitopes in Circulating Swine Influenza A Strains from 2013-2017.," *Vaccines*, vol. 9, no. 5, May 2021, doi: 10.3390/vaccines9050468.

[3] B. Reynisson, B. Alvarez, S. Paul, B. Peters, and M. Nielsen, "NetMHCpan-4.1 and NetMHCIIpan-4.0: improved predictions of MHC antigen presentation by concurrent motif deconvolution and integration of MS MHC eluted ligand data," *Nucleic Acids Res.*, vol. 48, no. W1, pp. W449–W454, Jul. 2020, doi: 10.1093/nar/gkaa379.

[4] A. H. Gutiérrez *et al.*, "T-cell epitope content comparison (EpiCC) of swine H1 influenza A virus hemagglutinin," *Influenza Other Respi. Viruses*, vol. 11, no. 6, pp. 531–542, Nov. 2017, doi: 10.1111/irv.12513.

[5] L. He, A. S. De Groot, A. H. Gutierrez, W. D. Martin, L. Moise, and C. Bailey-Kellogg, "Integrated assessment of predicted MHC binding and cross-conservation with self reveals patterns of viral camouflage.," *BMC Bioinformatics*, vol. 15 Suppl 4, no. Suppl 4, p. S1, 2014, doi: 10.1186/1471-2105-15-S4-S1.

[6] The UniProt Consortium, "UniProt: the universal protein knowledgebase in 2021," *Nucleic Acids Res.*, vol. 49, no. D1, pp. D480–D489, Jan. 2021, doi: 10.1093/nar/gkaa1100.

[7] A. S. De Groot *et al.*, "Better Epitope Discovery, Precision Immune Engineering, and Accelerated Vaccine Design Using Immunoinformatics Tools ," *Frontiers in Immunology* , vol. 11. p. 442, 2020, [Online]. Available: https://www.frontiersin.org/article/10.3389/fimmu.2020.00442.

[8] S. Southwood *et al.*, "Several common HLA-DR types share largely overlapping peptide binding repertoires.," *J. Immunol.*, vol. 160, no. 7, pp. 3363–3373, Apr. 1998.

[9] A. Sette and J. Sidney, "Nine major HLA class I supertypes account for the vast preponderance of HLA-A and -B polymorphism.," *Immunogenetics*, vol. 50, no. 3–4, pp. 201–212, Nov. 1999, doi: 10.1007/s002510050594.

[10] H. Arentz–Hansen *et al.*, "Celiac lesion T cells recognize epitopes that cluster in regions of gliadins rich in proline residues," *Gastroenterology*, vol. 123, no. 3, pp. 803–809, Sep. 2002, doi: 10.1053/gast.2002.35381.

[11] L. Moise *et al.*, "iVAX: An integrated toolkit for the selection and optimization of antigens and the design of epitope-driven vaccines," *Hum. Vaccin. Immunother.*, vol. 11, no. 9, pp. 2312–2321, Sep. 2015, doi: 10.1080/21645515.2015.1061159.

[12] M. S. A. Gilman, P. Furmanova-Hollenstein, G. Pascual, A. B. van 't Wout, J. P. M. Langedijk, and J. S. McLellan, "Transient opening of trimeric prefusion RSV F proteins," *Nat. Commun.*, vol. 10, no. 1, p. 2105, 2019, doi: 10.1038/s41467-019-09807-5.

[13] G. Taylor, “Animal models of respiratory syncytial virus infection.,” *Vaccine*, vol. 35, no. 3, pp. 469–480, Jan. 2017, doi: 10.1016/j.vaccine.2016.11.054.

[14] B. N. Blunck, W. Rezende, and P. A. Piedra, “Profile of respiratory syncytial virus prefusogenic fusion protein nanoparticle vaccine.,” *Expert Rev. Vaccines*, vol. 20, no. 4, pp. 351–364, Apr. 2021, doi: 10.1080/14760584.2021.1903877.

[15] N. I. Mazur *et al.*, “The respiratory syncytial virus vaccine landscape: lessons from the graveyard and promising candidates.,” *Lancet. Infect. Dis.*, vol. 18, no. 10, pp. e295–e311, Oct. 2018, doi: 10.1016/S1473-3099(18)30292-5.

[16] J. S. McLellan, W. C. Ray, and M. E. Peeples, “Structure and function of respiratory syncytial virus surface glycoproteins,” *Curr. Top. Microbiol. Immunol.*, vol. 372, pp. 83–104, 2013, doi: 10.1007/978-3-642-38919-1_4.

[17] G. Petrova, A. Ferrante, and J. Gorski, “Cross-reactivity of T cells and its role in the immune system,” *Crit. Rev. Immunol.*, vol. 32, no. 4, pp. 349–372, 2012, doi: 10.1615/critrevimmunol.v32.i4.50.

[18] X. Chen *et al.*, “Genetic variations in the fusion protein of respiratory syncytial virus isolated from children hospitalized with community-acquired pneumonia in China,” *Sci. Rep.*, vol. 8, no. 1, p. 4491, 2018, doi: 10.1038/s41598-018-22826-4.

[19] J. de Leeuw and P. Mair, “Multidimensional Scaling Using Majorization: SMACOF in R,” *J. Stat. Softw.*, vol. 31, no. 3 SE-Articles, pp. 1–30, Aug. 2009, doi: 10.18637/jss.v031.i03.

[20] V. N. Petrova and C. A. Russell, “The evolution of seasonal influenza viruses,” *Nat. Rev. Microbiol.*, vol. 16, no. 1, pp. 47–60, 2018, doi: 10.1038/nrmicro.2017.118.

[21] H. Chi, K.-L. Hsiao, L.-C. Weng, C.-P. Liu, and H.-F. Liu, “Persistence and continuous evolution of the human respiratory syncytial virus in northern Taiwan for two decades,” *Sci. Rep.*, vol. 9, no. 1, p. 4704, 2019, doi: 10.1038/s41598-019-41332-9.

[22] W. M. Sullender, “Respiratory syncytial virus genetic and antigenic diversity,” *Clin. Microbiol. Rev.*, vol. 13, no. 1, pp. 1–15, Jan. 2000, doi: 10.1128/CMR.13.1.1.

[23] J. Sidney, B. Peters, N. Frahm, C. Brander, and A. Sette, “HLA class I supertypes: a revised and updated classification.,” *BMC Immunol.*, vol. 9, p. 1, Jan. 2008, doi: 10.1186/1471-2172-9-1.

[24] N. Frahm *et al.*, “Extensive HLA class I allele promiscuity among viral CTL epitopes,” *Eur. J. Immunol.*, vol. 37, no. 9, pp. 2419–2433, Sep. 2007, doi: https://doi.org/10.1002/eji.200737365.

[25] B. Korber, M. LaBute, and K. Yusim, “Immunoinformatics comes of age,” *PLoS Comput. Biol.*, vol. 2, no. 6, pp. e71–e71, Jun. 2006, doi: 10.1371/journal.pcbi.0020071.
